# Incidental and Intentional Object–Location Memory: The Effects of Stimulant Treatment on Adults with ADHD

**DOI:** 10.3390/bs16091459

**Published:** 2026-08-22

**Authors:** Efrat Barel, Orna Tzischinsky, Shir Tahel Hai Tal

**Affiliations:** 1 Faculty of Psychology, College of Law & Business, Ramat Gan 5257346, Israel; 2 Department of Behavioral Sciences, The Max Stern Yezreel Valley College, Yezreel Valley 1930600, Israel; orna@yvc.ac.il (O.T.); shirtal612@gmail.com (S.T.H.T.)

**Keywords:** object–location memory, Attention Deficit/Hyperactivity Disorder, incidental encoding, intentional encoding

## Abstract

The relation between Attention Deficit/Hyperactivity Disorder (ADHD) and memory performance has attracted growing interest in recent years. However, one of the more complex memory abilities, object–location memory (OLM), remains scarcely studied within the context of ADHD. In this study, we examine incidental and intentional OLM performance in treated and untreated adults with ADHD and in controls. Fifty-eight adults with ADHD (26 medicated and 32 unmedicated) and 51 neurotypical individuals are assessed in a paper-and-pencil format for incidental and intentional OLM under a divided-attention condition. The results showed that all groups demonstrate higher performance in incidental OLM than in intentional OLM. Furthermore, medicated ADHD participants demonstrate reduced incidental OLM performance relative to unmedicated ADHD participants and controls. However, no group differences between unmedicated participants and controls are observed. This study reveals an overlooked aspect of visuospatial performance in ADHD under mental load and calls for further research on the impact of stimulants on incidental OLM.

## 1. Introduction

Attention Deficit/Hyperactivity Disorder (ADHD) is a neurodevelopmental disorder that persists across the lifespan, characterized by attention deficits, hyperactivity, and impulsivity (Goldstein & Goldstein, 1998). Numerous studies reveal impairments in adults with ADHD in various domains of cognition, including multiple aspects of attention (e.g., Fuermaier et al., 2022; Luna-Rodriguez et al., 2018), processing speed (e.g., Anker et al., 2022), executive functions (e.g., Tatar & Cansız, 2022), and memory (e.g., Pawley et al., 2024). In memory, the most acute impairments are found in verbal long-term memory (Hervey et al., 2004; Schoechlin & Engel, 2005), whereas studies on visuospatial memory in adults with ADHD are limited and yield inconsistent findings, with some showing group differences between ADHD and controls in favor of the latter (e.g., Gorohovsky & Magen, 2022) and others detecting no group differences (e.g., Skodzik et al., 2017). This inconsistency may be attributable to variance in domains and tests employed to measure visuospatial memory. One visuospatial memory ability that has been scarcely investigated in individuals with ADHD is object–location memory (OLM).

Object–location memory is a complex neurocognitive ability that challenges our cognitive system in a major way. Often considered a special class of episodic memory, it is frequently regarded as the pinnacle of human memory (Postma et al., 2008). Visuospatial memory is traditionally conceptualized as comprising distinct visual and spatial components: visual working memory operates to retain object properties such as forms, colors, and patterns, whereas spatial working memory encompasses dynamic, sequential mechanisms for spatial locations (Postma et al., 2008). Within this framework, OLM represents a specialized episodic memory domain. Rather than relying solely on individual visual or spatial processing, OLM uniquely requires the integration—or binding—of object identity (“what”) with its specific environmental coordinate (“where”) (Postma et al., 2008). Accordingly, OLM comprises three core components: object processing, spatial-location processing, and object–location binding. OLM is fundamental in our daily lives. Given its adaptive value for both humans and animals, it has been proposed that OLM is not solely driven by conscious recollections of object locations but rather functions as an automatic process potentially influenced by unconscious memory (Hasher & Zacks, 1979). Reck et al. (2010) examined group differences in laboratory-based object–location learning and memory tasks among boys (aged 7–12 years) and showed performance deficits among the ADHD group. In a similar vein, Gorohovsky and Magen (2022) showed that location memory for real-world objects is impaired in adults with ADHD in comparison with controls. In both studies, participants were explicitly instructed to memorize the location of objects, that is, to use *intentional* encoding.

The impact of attentional resources on encoding OLM has been extensively studied by contrasting performance across *incidental* and *intentional* encoding conditions. During intentional encoding, participants receive explicit advance notification about the required subsequent recall phase, whereas incidental encoding involves exposing participants to a stimulus array without any prior awareness of an impending retrieval requirement. Incidental and intentional encoding are also distinct in processing mechanisms: intentional encoding involves effortful processes whereas incidental encoding involves automatic processes (Hasher & Zacks, 1979). Findings regarding memory performance under both of these conditions are inconsistent. Some studies show that the locations of objects are learned without participants being instructed explicitly to remember the locations (e.g., Barel & Tzischinsky, 2020; Helbing et al., 2020). Other studies indicate that intention to remember locations improves memory performance (e.g., Naveh-Benjamin, 1988). Incidental and intentional memory performance among individuals with ADHD has not been contrasted directly. Studies incorporating both encoding conditions, however, suggest that participants with ADHD resemble controls in learning and memory patterns. Watanabe et al. (2010), for example, found that children with ADHD follow the same pattern of improvement in accuracy and speed as do neurotypical peers and resemble developmentally typical children in implicit and explicit visuomotor sequences. Taken together, although direct comparisons of incidental and intentional OLM in ADHD are lacking, the available evidence suggests that individuals with ADHD may exhibit learning patterns broadly comparable to those of neurotypical individuals across implicit and explicit learning contexts.

In an additional line of inquiry into incidental and intentional memory encoding, performance of individuals diagnosed with ADHD is compared with that of neurotypical individuals. While numerous studies have investigated memory performance of individuals with ADHD in intentional encoding conditions, few have addressed it under incidental encoding conditions, and they have yielded inconsistent findings. Gronchi and Peru (2022), for example, showed that unmedicated children with ADHD are less accurate and slower in incidental memory tasks than are developmentally typical peers. Ceci and Tishman (1984), in contrast, found that unmedicated children with ADHD outperformed their developmentally typical peers in an incidental-recognition task. To the best of our knowledge, the OLM performance of adults with ADHD has been investigated under intentional-encoding conditions (Gorohovsky & Magen, 2022) but not under incidental-encoding conditions. Furthermore, incidental and intentional OLM performance among treated and untreated adults with ADHD has not yet been studied at all.

Further attempts to characterize the cognitive challenges of individuals with ADHD focus on one of the most important executive functions: divided attention (Baddeley, 2002). In divided-attention tasks, participants are required to respond to both target and distractor stimuli. For decades, research has shown that memory encoding is typically impaired under divided attention as against full-attention conditions (e.g., Greene & Naveh-Benjamin, 2022). An important exception to this rule, however, is the attentional boost effect (ABE; Swallow & Jiang, 2010), in which memory of background or incidentally presented stimuli may remain intact—or even be enhanced—when encoded concurrently with a target-detection task. Indeed, various paradigms have demonstrated that simultaneous processing of background stimuli does not necessarily hamper verbal or visuospatial memory performance (e.g., Spataro et al., 2013) and in some cases yields a memory advantage (e.g., Prull, 2019; Spataro et al., 2025). Crucially, because divided attention differentially engages effortful versus automatic attentional resources, its interaction with encoding type may vary: while explicit, effortful strategies required during intentional encoding are highly susceptible to dual-task interference, automatic background encoding during incidental tasks may be preserved or modulated by attentional dynamics (Hasher & Zacks, 1979; Swallow & Jiang, 2010). Studies on divided attention with ADHD yield contradictory findings, some showing deficits in the ADHD group (e.g., Karatekin, 2004), others revealing no group differences (Inasaridze & Bzhalava, 2011), and yet others reporting that the ADHD group outperformed the control group (Elosúa et al., 2017). The role of attention during object–location long-term-memory encoding has hardly been studied (e.g., Barel, 2019). To the best of our knowledge, research on OLM under divided attention in people with ADHD remains unexplored. Therefore, in this study, we evaluate incidental and intentional OLM performance in medicated and unmedicated individuals with ADHD under divided-attention conditions. Given the contradictory findings regarding OLM performance in individuals with ADHD, we wish to precisely identify the conditions under which mental load impacts performance in both incidental and intentional memory encoding tasks. We achieve this by increasing mental demand and varying encoding conditions for participants with and without ADHD.

Another issue addressed in this study is the effect of long-term pharmacological treatment of ADHD (with stimulants including amphetamine and methylphenidate) on incidental and intentional OLM performance. Mechanistically, methylphenidate and amphetamines block dopamine and norepinephrine transporters, thus preventing catecholamine reuptake and boosting receptor availability—which, in turn, significantly modulates prefrontal attentional networks (MacDonald et al., 2024). Results from neuropsychological studies show that stimulants mainly improve vigilance performance. Their effects on more complex functions, however, are diverse and less consistent (Advokat, 2010). For example, medicated participants with ADHD significantly outperformed unmedicated participants with ADHD in the interference list on the Rey Auditory Verbal Learning Test (Pawley et al., 2024). Although no empirical research specifically investigates the role of pharmacological treatment on OLM in adults with ADHD, valuable insights may be drawn from the broader literature on visuospatial working memory and visual long-term memory. A recent meta-analysis revealed no significant difference in object-recognition memory between medicated and non-medicated groups (Lobato-Camacho & Faísca, 2024). In a review focusing on the effects of methylphenidate and amphetamines on cognition among adults with and without ADHD, Advokat (2010) concluded that stimulants do improve sustained attention but do not improve, and may impair, short-term acquisition of information and cognitive flexibility. Although the precise mechanisms that enable stimulants to influence cognitive performance remain to be fully elucidated, emerging evidence suggests that different cognitive functions require distinct dopamine levels. Specifically, optimal cognitive execution depends on a complex interplay among baseline dopaminergic tone, the specific task requirements, and the underlying neural circuitry (Cools & D’Esposito, 2011). When individuals perform complex visuospatial tasks under divided attention, stimulant-induced increases in prefrontal dopamine may induce a state of attentional narrowing or cognitive over-arousal (Ashinoff & Abu-Akel, 2021). Given that the effects of medication on memory performance and, especially, on OLM have not yet been established, the current study aims to address this issue.

Furthermore, it is consistently found in research that regular adequate sleep significantly enhances behavioral alertness and cognitive performance (Banks & Dinges, 2007). Specifically, prior research has demonstrated that sleep restriction and poor sleep quality significantly impair spatial memory and object–location memory performance (e.g., Van Der Werf et al., 2009; Barner et al., 2023). Given that sleep disturbances are highly prevalent among individuals with ADHD and may compromise visuospatial binding and attentional networks independently, in this study, we monitored sleep objectively and subjectively before testing by measuring sleep quality and quantity during the four nights preceding the experiment. This was done to ensure that participants had not suffered from sleep deprivation in the nights preceding the study and that the study groups did not differ in sleep measures.

In sum, to the best of our knowledge, OLM performance under cognitive load among ADHD participants has not yet been investigated. We address this lacuna among young adults diagnosed with ADHD—half undergoing medication, half not—and a third group of young adults with no ADHD diagnosis as a control group. Given the inconsistent findings on the effectiveness of pharmacological treatment for adults with ADHD (Advokat, 2010), we aim to deepen our understanding of an understudied area and focus on OLM performance in treated and untreated adult individuals with ADHD and in controls. Finally, the study controls for the quality and quantity of sleep, which has been found to interact with cognitive performance.

Basing ourselves on the reviewed literature, we formulated two hypotheses. First, ADHD participants resemble controls in their pattern of OLM performance under divided attention, demonstrating superior incidental relative to intentional encoding performance across all groups. This pattern would be consistent with the automaticity hypothesis. Second, we asked whether OLM performance differs among medicated ADHD participants, unmedicated ADHD participants, and controls; given the limited and inconsistent literature, we formulated no directional hypothesis regarding group differences.

## 2. Materials and Methods

### 2.1. Participants

Our sample comprised 109 participants (53 males and 56 females; mean age 23.9 ± 2.76) from a college in northern Israel. Fifty-eight participants were diagnosed with ADHD; 51 (the control group) were not. Twenty-six members of the ADHD group were undergoing medical treatment for ADHD (hereinafter: the ADHD-medicated group). The remaining 32 were not undergoing medical treatment for ADHD (ADHD-unmedicated group). The three groups were differentiated neither by age [*F*(2, 106) = 0.61, *p* > 0.05] nor by sex [χ^2^(2) = 0.53, *p* > 0.05]. Participants were recruited through advertisements at the college and received course credit for their participation. Participants in the ADHD groups were required to present documentation of a previous ADHD diagnosis made by a psychiatrist or neurologist. Our research assistant verified that the diagnosis had been established by a qualified physician. ADHD diagnosis in Israel follows internationally accepted diagnostic criteria based on the DSM framework; however, the specific diagnostic criteria documented for each participant were not available to the research team. To protect participants’ privacy, copies of the diagnostic reports were not retained. Each member of the ADHD-medicated group received a dosage of medication: methylphenidate (62% of medicated ADHD participants); mixed amphetamine salt, or dexamphetamine (38% of medicated ADHD participants—see Supplementary Table S1) that was adjusted to their weight, age, and clinical situation, with a mean treatment duration of four years (SD = 4.36). All participants in the medicated ADHD group completed the cognitive assessment while taking their prescribed stimulant medication. However, the exact timing of the last dose on the day of testing was not recorded. Consequently, whether testing occurred within the medication’s active therapeutic period could not be verified. Given the small number of participants who received each medication, medication type and dosage were not entered as covariates in the statistical analyses. Exclusion criteria were applied to the three groups and included psychiatric diagnoses other than ADHD and medical or neurological disorders.

### 2.2. Materials and Procedure

The study was approved by the institutional review board. All participants arrived at the lab between 8:00 a.m. and 12:00 p.m., after four nights of objective sleep recording by ActiGraph (Mini Act, AMA-32, Ambulatory Monitoring, Inc., Ardsley, NY, USA), to participate in the experiment. After giving their informed consent, participants completed a brief demographic questionnaire, the subjective measurement of sleepiness, and the OLM tasks. The measures and tasks are outlined below.

#### 2.2.1. Object–Location Memory—Incidental Encoding

In this task, participants viewed an array of 25 black-and-white drawings of objects, adapted from stimuli used in Eals and Silverman’s (1994) study. The items were printed on standard A4 white paper. Participants were instructed to perform two tasks—a pricing task and a distraction task—within a one-minute period. The pricing task served as the manipulation for incidental (non-explicit) encoding. Participants were instructed to assign a price tag to each object directly on the paper. If a precise price estimation was not feasible, they were told to provide an educated guess (Eals & Silverman, 1994). The distraction task was integrated to elevate cognitive load; this involved the presentation of a pre-recorded soundtrack featuring piano tones randomized by pitch (low or high) at intervals of two or three seconds (Palmer et al., 2013). Participants indicated low tones by raising their left hand and high tones by raising their right hand. Immediately following the encoding phase, participants were presented with a second stimulus array. In this array, 14 of the objects had been relocated. They were given 60 s to identify and mark the objects whose locations had remained constant and circle those that had been displaced. A manipulation check confirmed that the participants were not aware of the true purpose of the study. Immediately following the incidental-encoding phase, a structured manipulation check was administered to confirm that participants remained unaware of the memory evaluation. The research assistant asked each participant: “*What do you think is the true purpose of this study?*” According to a predefined protocol, any participant who correctly identified the study as being aimed at assessing object–location memory was to be excluded from the sample. In the current study, no participants identified the true memory objective, confirming that the incidental-encoding manipulation was effective across all participants. A memory score corresponding to the number of correctly detected location-exchanged objects was generated. A tone-discrimination score for the distraction task was recorded as well. (A correct answer was scored 1, and all scores were totaled.)

#### 2.2.2. Object–Location Memory—Intentional Encoding

The intentional-encoding condition paralleled the incidental task in structure and materials but employed a distinct stimulus array. The key procedural difference was that participants were explicitly instructed to dedicate one minute to “try to memorize as many objects in the array as possible and their approximate locations” (Eals & Silverman, 1994, p. 100) before introducing the distraction task.

The paper-and-pencil format was deliberately selected for the current study due to the unique demands imposed by the incidental-encoding task. Participants were required to write a price estimate for each item, and given the brief time frame (60 s), as well as variation in participants’ computer proficiency, a paper-and-pencil format was considered more appropriate. This approach has also been used in previous research employing similar procedures (e.g., Barel, 2019).

#### 2.2.3. Sleep Patterns

The objective sleep assessment was performed to monitor participants’ sleep quality and quantity to ensure that participants were not suffering from sleep deprivation in the four days preceding the study.

Subjective sleepiness was measured on the Karolinska Sleepiness Scale (KSS) (Åkerstedt & Gillberg, 1990), a self-assessment questionnaire that measures subjective sleepiness. Participants rated this metric from extremely alert (1) to very sleepy, struggling to stay awake (9). The KSS has been validated against objective sleepiness measurements using EEG, and significant correlations have been found between objective and subjective assessments. Participants completed the KSS questionnaire in the morning before the study procedure.

### 2.3. Procedure

Participants were recruited via advertisements on the college campus. Our research assistant met with potential participants at the psychology lab on campus. After giving informed consent in writing, participants were required to maintain a regular sleep pattern during the days of the study. Subjective sleepiness was measured using the KSS. Objective sleep patterns were monitored by means of an ActiGraph (AMI, New York, NY, USA), a small device that measures sleep patterns in one’s natural environment and yields objective data on them. Participants wore the ActiGraph continuously during the four nights prior to the study. The resulting recordings provided estimates of several sleep parameters, including sleep onset, wake time, sleep latency, total sleep duration, minutes spent asleep, wake after sleep onset (WASO), and overall sleep efficiency.

All participants were tested in the same room in the lab. Due to the study design, which included an incidental-encoding condition, the memory tasks were administered to all participants in a fixed sequence, with the incidental task always preceding the intentional one.

### 2.4. Data Analysis

All statistical analyses were performed using SPSS version 28.0. Descriptive statistics are presented as mean ± standard deviation. Missing data were minimal (one missing value; <1%) and were handled using available-case analysis. Given the negligible proportion of missing data, no imputation procedure was applied. The Kolmogorov–Smirnov test was used to assess the normality of data distributions. Where a skewed distribution was found, variables were either log-transformed or transformed by taking their square root. Homogeneity of variance across groups was verified using Levene’s test, which yielded non-significant results for all primary memory outcome variables (*p >* 0.05).

We performed a two-way mixed analysis of variance (ANOVA) on two independent variables—encoding type (incidental, intentional) and group (no ADHD, ADHD-unmedicated, ADHD-medicated) and the OLM score as the dependent variable. Following the significant encoding type × group interaction, simple-effects comparisons were conducted to compare incidental and intentional OLM performance within each of the three groups. A Bonferroni correction was applied to these three planned comparisons (α = 0.05/3 = 0.017). For the one-way ANOVAs examining group differences separately in incidental and intentional OLM, Tukey’s HSD procedure was used for all pairwise group comparisons, as it controls the family-wise error rate across multiple pairwise comparisons. Tukey-adjusted *p*-values were evaluated at α = 0.05. Because no a priori power analysis was conducted, a sensitivity analysis was performed using G*Power 3.1.9.7 to evaluate the statistical power afforded by the available sample. For the one-way ANOVA comparing the three study groups (*n* = 109), with α = 0.05 and power set at 0.80, the analysis indicated a minimum detectable effect size of f = 0.30. Thus, the study was sufficiently powered to detect approximately medium or larger omnibus group effects.

## 3. Results

### 3.1. Sleep and OLM Measures

Means and standard deviations for all study variables are shown in Table 1. The objective measures of sleep demonstrated that the participants’ sleep characteristics adhered to the normative standards; specifically, sleep duration was consistent with recommended amounts, and sleep quality was evaluated as high.

### 3.2. Group Differences in Sleep Measures and in the Distraction Task

Supplementary Table S2 presents correlations among incidental and intentional OLM and sleep measures. None of the correlations were significant (all *p*s > 0.05).

Furthermore, three one-way ANOVAs were conducted to examine group differences in sleep efficiency, sleep duration, and subjective sleepiness (KSS). No significant group differences were found for any of the three sleep measures (all *p*s > 0.05; see Supplementary Table S3). Because sleep parameters were homogeneous across groups and showed no association with memory performance, we did not include them as covariates.

One-way ANOVAs were also conducted, with group (ADHD-unmedicated, ADHD-medicated, and control) as the independent variable and the tone-discrimination score as the dependent variable. Group differences were found neither under the incidental condition [*F*(2, 106) = 0.02, *p* > 0.05; *η*^2^ = 0.00] nor under the intentional condition [*F*(2, 106) = 0.97, *p* > 0.05; *η*^2^ = 0.02].

### 3.3. Incidental vs. Intentional Memory Performance

**Figure 1:**
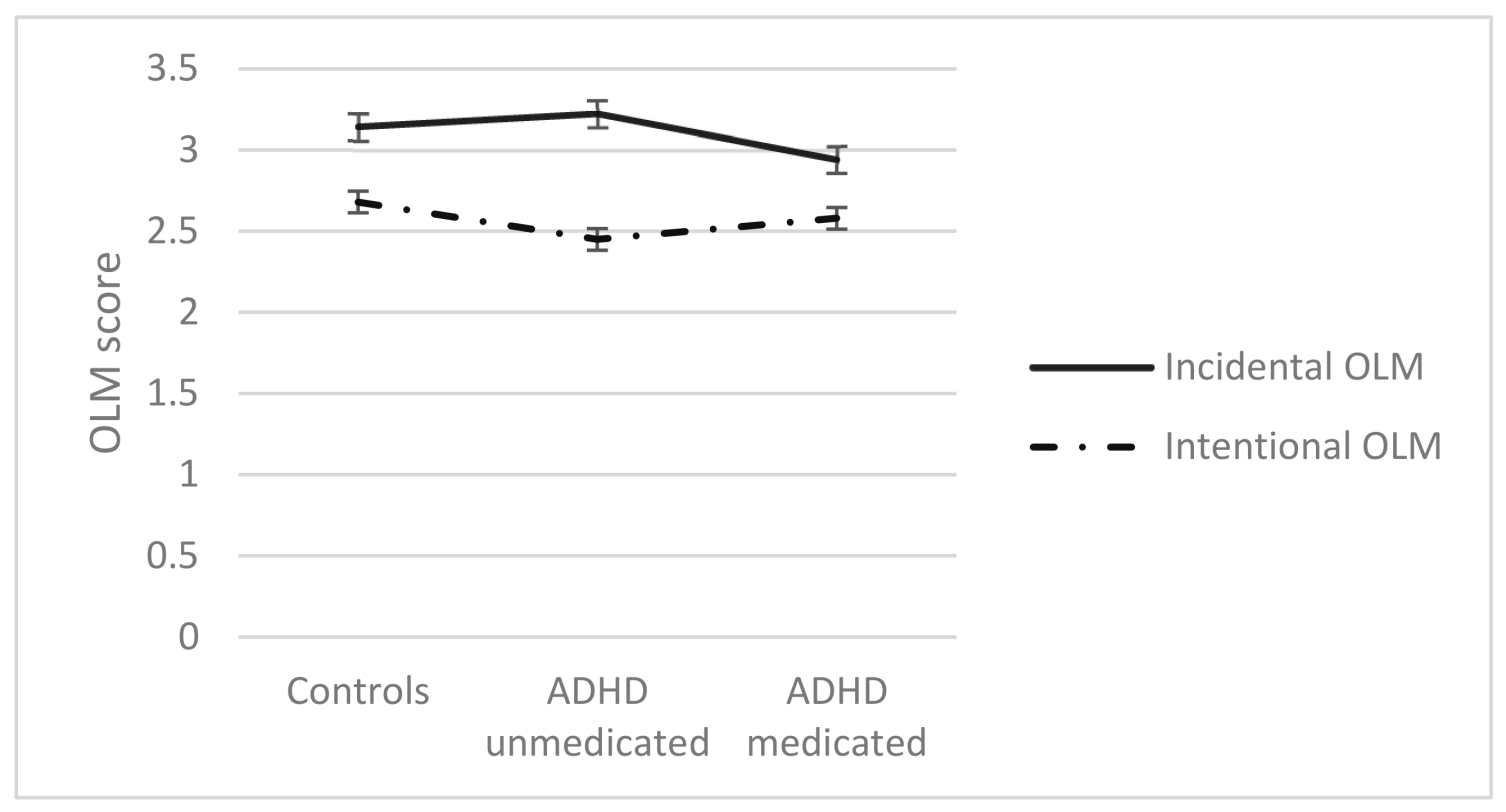
Estimated marginal means (±SE) of square-root-transformed OLM scores under incidental and intentional encoding across the three study groups. Notes: OLM = object–location memory; ADHD = Attention Deficit/Hyperactivity Disorder.

We performed a two-way mixed ANOVA with two independent variables—encoding type (incidental or intentional) and group (no ADHD, ADHD-unmedicated, or ADHD-medicated)—and memory score as the dependent variable. A significant main effect was found for encoding type [*F*(1, 105) = 91.60, *p* < 0.001; *η*^2^ = 0.47, 95% CI 0.33, 0.57]. Participants scored higher in OLM in incidental encoding than in intentional encoding. The main effect for group was non-significant [*F*(2, 105) = 2.35, *p* > 0.05; *η*^2^ = 0.04, 95% CI 0.02, 0.13]. However, the interaction encoding type × group was significant [*F*(2, 105) = 4.11, *p* < 0.05; *η*^2^ = 0.07, 95% CI 0.001, 0.17]. By decomposing the interaction (with Bonferroni adjustment), we found that participants in each group scored higher in OLM in incidental encoding than in intentional encoding, with the largest effect size obtained for the ADHD-unmedicated group (Cohen’s *d* = 1.48, 95% CI 0.97, 1.98), followed by the no-ADHD group (Cohen’s *d* = 0.96, 95% CI 0.62, 1.29) and lastly the ADHD-medicated group (Cohen’s *d* = 0.51, 95% CI 0.01, 0.91). (See Figure 1).

### 3.4. Group Differences in Incidental and Intentional Memory Performance

Incidental and intentional OLM performance was compared across the three groups of participants in a one-way analysis of variance. The results are presented in Table 2. Group differences were found for incidental-OLM memory score. Tukey’s post hoc tests revealed that the ADHD-medicated group obtained lower memory scores on incidental OLM than did the ADHD-unmedicated and the control groups. Complete pairwise group comparisons, including Tukey-adjusted *p*-values, 95% confidence intervals, and effect sizes, are presented in Supplementary Table S4.

## 4. Discussion

In this study, we investigated the OLM performance of medicated and unmedicated ADHD participants under mental load. Our first aim was to explore memory patterns in ADHD participants. In the context of memory performance under incidental and intentional encoding conditions, research on spatial memory has been marked by longstanding debates. Proponents of the “automaticity hypothesis” argue that the encoding of object locations may occur even in the absence of attention allocation (Hasher & Zacks, 1979), whereas other studies have demonstrated that participants’ awareness of an upcoming retrieval task may enhance performance (e.g., Naveh-Benjamin, 1988). Our findings are consistent with the automaticity hypothesis by demonstrating that participants across all groups in the study performed better under incidental than under intentional memory conditions. Although research has shown that the intention to learn locations may enhance memory performance, our findings indicate that participants were able to encode locations without being explicitly instructed to do so.

The study employed a within-subject design, with each participant completing both the incidental and intentional conditions. This paper-and-pencil approach has been adopted in previous research, including two studies that used the same format, one conducted as a between-subject design (Barel, 2019) and the other as a within-subject design (Barel & Tzischinsky, 2020), reporting similar results: stronger performance under the incidental condition than under the intentional condition. Furthermore, in the present study, participants encoded object locations while under attentional load as they were required to engage simultaneously in an auditory task. Despite the need to allocate attention to this additional task, their ability to encode object locations in the incidental condition remained intact, indicating that the distraction task did not exhaust their attentional resources. The emergence of resemblance between the groups in our study in regard to OLM performance patterns under encoding conditions supports previous findings regarding learning and memory patterns. Karatekin et al. (2009) found that ADHD and control groups did not differ in incidental and intentional spatial sequence learning. Although our ADHD groups did show some accuracy impairments, they showed a similar learning effect across manual and oculomotor modalities. These findings suggest that incidental object–location encoding remained relatively robust under divided attention, a pattern consistent with the automaticity hypothesis despite increased cognitive demands.

We also examined group differences in incidental and intentional OLM under mental load. We found that the ADHD-medicated group attained lower memory scores on incidental OLM than did the ADHD-unmedicated and the control groups. These findings may appear inconsistent with the well-established clinical benefits of stimulant medication in adult ADHD. Contemporary evidence indicates that stimulant treatment effectively attenuates core ADHD symptoms, improves everyday functioning, and is associated with lower risks of adverse functional outcomes. At the cognitive level, stimulant medication has also been shown to improve several executive functions, particularly attention, inhibition, working memory, and processing speed (Gonda et al., 2026; Isfandnia et al., 2024). However, considerably less is known about the effects of stimulant medication on long-term visuospatial memory, and evidence regarding OLM is virtually absent. Several animal models of ADHD show differentiated effects of stimulants on various spatial memory functions. Bouchatta et al. (2018), for example, used the 6-hydroxydopamine injection lesioned mouse model, whereas Miguel et al. (2020) used the neonatal hypoxia-ischemic model in rats. In both studies, stimulant administration impaired object recognition. With regard to human studies, a recent meta-analysis revealed a non-significant difference in object-recognition memory between medicated and non-medicated groups (Lobato-Camacho & Faísca, 2024), whereas Advokat (2010), in a review focusing on the effects of methylphenidate and amphetamines on cognition among adults with and without ADHD, concluded that stimulants do not improve and may even impair short-term acquisition of information and cognitive flexibility. To the best of our knowledge, no previous study examined the role of stimulants on incidental and intentional OLM performance. Our findings suggest that stimulant treatment was associated with lower OLM performance under mental load among ADHD participants than among both unmedicated ADHD participants and controls. However, further studies examining associations between medication status and visuospatial performance are still needed. Visuospatial memory has been studied through various paradigms, assessments, materials, and tasks. Diaz-Orueta et al. (2022), in their critical review, outlined several directions of criticism revolving mainly around the broad availability of tests that assess visuospatial memory, which, in fact, deal with some important challenges. Our preliminary findings further support the view that spatial memory performance is not a unitary function, highlighting the need for further studies to examine whether OLM performance varies according to stimulant type, dosage, and treatment context.

Our ADHD-unmedicated group did not differ from our control group in both incidental and intentional OLM. Although executive-function weaknesses are well documented at the group level in ADHD, they are not uniformly expressed across individuals or cognitive domains. Studies of adults with ADHD demonstrate substantial neuropsychological heterogeneity, with some individuals showing broadly intact cognitive performance and cognitive subgroups extending across ADHD and control samples (Mostert et al., 2015, 2018). Thus, the absence of group differences in the present OLM task may reflect the possibility that an ADHD diagnosis may not entail impairment in every executive or memory process. As for location-memory tasks, previous studies yielded inconsistent findings. For example, Mariani and Barkley (1997) observed a deficit in spatial working memory in unmedicated children with ADHD in a task assessing the memory of the spatial location of a picture. In contrast, in Karatekin’s (2004) study, participants were asked to remember the locations of letters presented on a screen. No differences were found between unmedicated children with ADHD and controls in this spatial-memory task. Studies on OLM in adults with and without ADHD are limited. In Gorohovsky and Magen’s (2022) study, university students with and without ADHD were asked to detect change in token or orientation of real-world objects in natural scenes. The authors found that the memory of individual objects was impaired in the ADHD group in comparison with controls. To the best of our knowledge, long-term OLM has not yet been investigated among ADHD participants. In their meta-analysis, Skodzik et al. (2017) found that adults with ADHD did not differ from their counterparts on visual long-term memory. They also reported an association of long-term memory deficits with memory-acquisition deficits but not with retrieval problems, suggesting that visual long-term memory deficits in adults with ADHD reflect a learning impairment at the encoding stage. Studies that probed the neural correlates of visuospatial memory in ADHD and healthy controls suggest that ADHD participants may demonstrate performance comparable to that among controls but may do so less efficiently. For example, Bédard et al. (2014) found that unmedicated ADHD participants and controls performed comparably on a visuospatial working-memory task (the n-back test, in which participants have to determine if a specific visuospatial stimulus has already been presented). ADHD participants, however, showed bilateral activation in the left dorsolateral prefrontal cortex (DLPFC) and the posterior cingulate cortex (PCC) and reduced left DLPFC and PCC connectivity relative to controls for high-load memory. The authors asserted that ADHD participants need to exert greater neural and mental effort to achieve a normal level of performance under mental load. Moreover, OLM is a multicomponent ability involving object processing, spatial-location processing, and object–location binding (Postma et al., 2008); therefore, it should not be regarded as a direct proxy for executive functioning. The relatively well-preserved performance of the unmedicated ADHD group may indicate that the specific processes required by the present task were sufficiently intact, or that these participants were able to mobilize alternative strategies or additional cognitive resources to maintain performance. Given previous studies that showed neural differences between ADHD participants and healthy controls in visuospatial working-memory performance, further investigation of the neural basis for OLM performance with and without mental load among ADHD participants is desiderata. Future neuroimaging studies should examine whether preserved OLM performance in unmedicated adults with ADHD is supported by compensatory activation or altered connectivity within prefrontal and visuospatial memory networks. Our findings suggest that intact behavioral performance should not necessarily be interpreted as evidence of intact underlying cognitive or neural mechanisms in adults with ADHD.

This study has several limitations. First, it investigated incidental and intentional OLM performance under mental load among ADHD participants. Future studies should deepen our understanding of group differences under various conditions, such as full versus divided or selective attention. Second, OLM paradigms vary in the retention time they employ, from a few seconds up to minutes. Although object–location memory supports a variety of everyday activities, the present study assessed this ability using a controlled paper-and-pencil paradigm with immediate recall. Although standardized neuropsychological memory tests have demonstrated meaningful associations with everyday memory functioning (Chaytor & Schmitter-Edgecombe, 2003), the ecological validity of the present findings remains limited by the controlled nature of the task. Future studies should therefore strengthen ecological validity by employing more naturalistic paradigms and longer retention intervals. Third, although the sensitivity analysis indicated that the overall sample was sufficient to detect approximately medium or larger omnibus group effects, statistical power to detect smaller effects, interactions, and pairwise group differences may have been limited, particularly given the relatively small medicated ADHD group (*n* = 26). The size of this subgroup also precluded examination of the differentiated effects of medication type, dosage, and treatment duration. Future studies with larger medicated samples are therefore needed to detect more subtle group differences and examine potential heterogeneity in treatment effects. An additional limitation concerns the lack of a direct measure of ADHD symptom severity. Participants receiving pharmacological treatment may have represented individuals with more severe clinical presentations, as medication is often prescribed for those experiencing greater functional impairment. Consequently, the poorer incidental OLM performance observed in the medicated ADHD group cannot be attributed solely to medication status. Future studies should include standardized measures of ADHD symptom severity to disentangle the potential contributions of symptom severity and pharmacological treatment to memory performance. Furthermore, although all medicated participants were undergoing ongoing stimulant treatment, the timing of the last dose on the day of testing was not recorded. Therefore, we could not verify whether each participant was tested during the medication’s active therapeutic period. Moreover, because medication status was not randomized and the medicated group was heterogeneous with respect to stimulant type and dosage, the observed group differences should be interpreted as associations with treatment status rather than as evidence of causal medication effects. Another methodological consideration is the fixed administrative order, in which the incidental-encoding task always preceded the intentional-encoding task. This rigid sequence was procedurally necessary within our within-subject design to preserve the validity of the incidental condition, as prior awareness of a memory test would have converted incidental encoding into an explicit and intentional strategy. Nevertheless, this fixed order introduces potential order effects such as practice, fatigue, or carryover effects, which may have influenced intentional OLM performance. Consequently, the observed differences between the incidental and intentional conditions cannot be attributed exclusively to encoding type. To overcome this constraint and validate these findings, future studies should employ a between-subjects design, allowing encoding conditions to be counterbalanced across separate participant cohorts. Future longitudinal studies and, where feasible, randomized medication trials are needed to better disentangle the causal effects of stimulant treatment from pre-existing differences in ADHD symptom severity and cognitive functioning.

## 5. Conclusions

This study explored participants with and without ADHD (medicated and unmedicated) in incidental and intentional OLM under a divided-attention condition. The results showed that all groups demonstrated higher performance in incidental OLM than in intentional OLM, a pattern consistent with the automaticity hypothesis. Indeed, Postma et al. (2008) speculated that each component of object–location memory differs in its processing automaticity, with the spatial-location component operating more automatically than the object identity and object–location binding processing components. Furthermore, we found that medicated ADHD participants demonstrated lower incidental OLM performance compared with unmedicated ADHD participants and controls. Previous studies using animal models of ADHD suggest that stimulant administration was associated with poorer object recognition performance. Furthermore, the lack of difference between unmedicated ADHD participants and controls conforms with previous studies suggesting that, although there are well-established differences between these groups in vigilance performance and several executive functions, they do not differ in other memory skills. Studies investigating the neural correlates of visuospatial memory in ADHD suggest that ADHD participants may not differ from neurotypical controls in performance but show less prefrontal efficiency than do controls. Therefore, the present findings provide preliminary evidence of differences in incidental OLM performance across treatment-status groups of adults with ADHD.

## Figures and Tables

**Table 1:** Sleep and OLM measures.

	Mean	SD
Sleep efficiency (%)	95.57	2.88
Sleep duration (h)	7.09	0.90
KSS	4.30	1.91
Incidental OLM	8.01	2.51
Intentional OLM	4.92	2.38

Note: KSS—Karolinska Sleepiness Scale; OLM—object–location memory.

**Table 2:** Group differences in incidental and intentional OLM during divided attention.

	Controls		ADHD Unmedicated		ADHD Medicated		*F*	df	*η* ^2^	*CI*
	*M*	*SD*	*M*	*SD*	*M*	*SD*				
Incidental OLM	8.26	2.27	8.53	2.43	6.88	2.80	4.08 *	2, 105	0.07	0.001, 0.17
Intentional OLM	5.37	2.42	4.19	2.10	4.92	2.51	1.85	2, 106	0.04	0.000, 0.12

Notes: OLM = object–location memory; ADHD = Attention Deficit/Hyperactivity Disorder. * *p* < 0.05. Value in boldface represents the comparison that remained statistically significant after application of Bonferroni correction for multiple comparisons (α = 0.05/2 = 0.025). Means and standard deviations are presented for the original scale scores. Inferential statistics for OLM were computed using square-root-transformed OLM scores.

## Data Availability

The datasets are available upon request. The raw data supporting the conclusions of this article will be made available by the authors, without undue reservation.

## Supplementary Materials

The following supporting information can be downloaded at https://www.mdpi.com/article/10.3390/bs16091459/s1: Table S1. ADHD medication characteristics. Table S2. Correlation between OLM scores and sleep measures. Table S3. Group differences (controls, ADHD-unmedicated, ADHD-medicated) in sleep measures. Table S4. Pairwise group comparisons of incidental and intentional object–location memory performance.

## Abbreviations

The following abbreviations are used in this manuscript:

ADHD	Attention Deficit/Hyperactivity Disorder
OLM	Object–location memory
KSS	Karolinska Sleepiness Scale
ANOVA	Analysis of variance
DLPFC	Dorsolateral prefrontal cortex
PCC	Posterior cingulate cortex
