# Supplementary material for: Incidental and Intentional Object–Location Memory: The Effects of Stimulant Treatment on Adults with ADHD
Source: Behav Sci (Basel). 2026 Aug 22;16(9):1459. doi: 10.3390/bs16091459 (PMC13603106; doi:10.3390/bs16091459)
Supplement: Supplementary file 1 [file behavsci-16-01459-s001.zip › behavsci-4373055-supplementary.pdf]

Table S1. ADHD medication characteristics

Medication	Dose ranges (mg/day)
Attent	10-15
Ritalin	20-40
Concerta	36
Vyvanse	Not available

Note. ADHD = Attention Deficit/Hyperactivity Disorder.

Table S2. Correlation between OLM scores and sleep measures.

	Sleep efficiency	Sleep duration	KSS
**Incidental memory**	0.06	0.08	-0.02
**Intentional memory**	0.15	-0.01	0.08

Note. OLM = Object location memory; KSS = the Karolinska Sleepiness Scale.

Table S3. Group differences (controls, ADHD-unmedicated, ADHD-medicated) in sleep measures.

	F	df	p	η2	CI
**Sleep efficiency**	0.59	2,105	.558	.01	.00, .07
**Sleep duration**	0.07	2,105	.937	.00	.00, .02
**KSS**	0.55	2,105	.576	.01	.00, .06

Note. ADHD = Attention Deficit/Hyperactivity Disorder; KSS = the Karolinska Sleepiness Scale; CI = confidence interval

Table S4. Pairwise Group Comparisons of Incidental and Intentional Object–Location Memory Performance

OLM condition	comparison	Mean difference	Tukey-adjusted p	CI	Cohen's d
**Incidental encoding**					
	ADHD medicated - ADHD unmedicated	-0.28	.027	-0.54,-0.03	0.61
	ADHD medicated - controls	-0.25	.039	-0.48,-0.01	0.54
	ADHD unmedicated - controls	0.04	.911	-0.18,0.26	0.11
**Intentional encoding**					
	ADHD medicated - ADHD unmedicated	0.13	.528	-0.16,0.42	0.28
	ADHD medicated - controls	-0.09	.698	-0.36,0.18	0.22
	ADHD unmedicated - controls	-0.22	.089	-0.47,0.03	0.54

Note. OLM = object location memory; ADHD = Attention Deficit/Hyperactivity Disorder; CI = confidence interval.
